# A Web-Based Tool to Support Shared Decision Making for People With a Psychotic Disorder: Randomized Controlled Trial and Process Evaluation

**DOI:** 10.2196/jmir.2851

**Published:** 2013-10-07

**Authors:** Lian van der Krieke, Ando C Emerencia, Nynke Boonstra, Lex Wunderink, Peter de Jonge, Sjoerd Sytema

**Affiliations:** ^1^University of GroningenUniversity Medical CenterUniversity Center for PsychiatryGroningenNetherlands; ^2^University of GroningenJohann Bernoulli Institute for Mathematics and Computer ScienceGroningenNetherlands; ^3^Friesland Mental Health Care ServiceLeeuwardenNetherlands

**Keywords:** psychotic disorders, schizophrenia, computers, computer-assisted decision making, shared decision making, feasibility studies, randomized clinical trial

## Abstract

**Background:**

Mental health policy makers encourage the development of electronic decision aids to increase patient participation in medical decision making. Evidence is needed to determine whether these decision aids are helpful in clinical practice and whether they lead to increased patient involvement and better outcomes.

**Objective:**

This study reports the outcome of a randomized controlled trial and process evaluation of a Web-based intervention to facilitate shared decision making for people with psychotic disorders.

**Methods:**

The study was carried out in a Dutch mental health institution. Patients were recruited from 2 outpatient teams for patients with psychosis (N=250). Patients in the intervention condition (n=124) were provided an account to access a Web-based information and decision tool aimed to support patients in acquiring an overview of their needs and appropriate treatment options provided by their mental health care organization. Patients were given the opportunity to use the Web-based tool either on their own (at their home computer or at a computer of the service) or with the support of an assistant. Patients in the control group received care as usual (n=126). Half of the patients in the sample were patients experiencing a first episode of psychosis; the other half were patients with a chronic psychosis. Primary outcome was patient-perceived involvement in medical decision making, measured with the Combined Outcome Measure for Risk Communication and Treatment Decision-making Effectiveness (COMRADE). Process evaluation consisted of questionnaire-based surveys, open interviews, and researcher observation.

**Results:**

In all, 73 patients completed the follow-up measurement and were included in the final analysis (response rate 29.2%). More than one-third (48/124, 38.7%) of the patients who were provided access to the Web-based decision aid used it, and most used its full functionality. No differences were found between the intervention and control conditions on perceived involvement in medical decision making (COMRADE satisfaction with communication: F_1,68_=0.422, *P*=.52; COMRADE confidence in decision: F_1,67_=0.086, *P*=.77). In addition, results of the process evaluation suggest that the intervention did not optimally fit in with routine practice of the participating teams.

**Conclusions:**

The development of electronic decision aids to facilitate shared medical decision making is encouraged and many people with a psychotic disorder can work with them. This holds for both first-episode patients and long-term care patients, although the latter group might need more assistance. However, results of this paper could not support the assumption that the use of electronic decision aids increases patient involvement in medical decision making. This may be because of weak implementation of the study protocol and a low response rate.

**Trial Registration:**

Dutch Trial Register (NTR) trial number: 10340; http://www.trialregister.nl/trialreg/admin/rctsearch.asp?Term=10340 (Archived by WebCite at http://www.webcitation.org/6Jj5umAeS).

## Introduction

Shared decision making in mental health care has been dubbed an ethical imperative [1]. Since the rise of recovery-oriented medicine, patients have been acknowledged as experiential experts and equal partners in communication with clinicians. Research has shown that people with severe and persistent mental disorders are no exception. People with psychotic disorders are able and willing to participate in medical decision making [2,3]. However, the desire for participation is greater than the amount of participation they actually experience [4,5]. A range of obstacles hamper successful implementation. Most clinicians believe in the benefits of shared decision making, but time constraints and a large number of clinical responsibilities prevent them from practicing it [6,7]. Moreover, patients may not be used to actively participating in medical decision making and they can lack access to medical information that is easily intelligible [8].

Drake and Deegan [9] stressed the need for decision aids and support centers to ensure the development of an infrastructure that facilitates the practice of shared decision making. Several initiatives have been developed in this area. For instance, in Germany, Hamann et al [3] investigated the effectiveness of a shared decision-making intervention with a printed decision aid for inpatients with schizophrenia. They found that patients using the decision aid had better knowledge about their disease and had a higher perceived involvement in medical decisions compared to a control group that received care as usual [3]. Recently, a special case was made for electronic decision aids [10] because they have various advantages over paper-based decision aids, such as presenting personalized information based on smart algorithms. So far, 3 electronic decision aids have been developed and investigated to support shared decision making in the treatment planning for people with severe mental disorders, but the results are inconsistent [11,12]. A pilot study by Deegan et al [11] showed that outpatients were able to work with a Web-based program to support shared decision making in psychopharmacological consultation. Patients used the program on computers at the clinic where experiential experts were available for assistance. Two small-scale randomized clinical trials were conducted [12,13]. The first trial showed that patients were able to electronically design their own care plan, but there was no difference between intervention and control groups in satisfaction with the care planning process, which was the primary outcome [12]. The second trial reported that a Web-based support system encouraging patients to discuss their current status and treatment with their clinician resulted in patients being more verbally active during health visits [13].

More evidence is needed to determine whether electronic decision aids are helpful in clinical practice and can lead to increased patient involvement and better outcomes. In addition, more information is needed about what proportion of patients are willing and able to work with Web-based decision aids and in what form (with or without assistance, using their own computer or a clinic computer). This paper reports on a randomized controlled trial and process evaluation of a Web-based intervention to facilitate shared decision making, with or without assistance, for people with psychotic disorders. Our aim was to investigate this intervention in a naturalistic setting, meaning that all eligible patients were included to be able to determine how many of them would actually use the decision aid.

## Methods

### Ethical Considerations

Informed consent was obtained by research nurses. Patients were provided with an information brochure and they received a phone number and email address of a research assistant who they could contact for further information. The national Dutch medical ethical committee for mental health care (Medisch-ethische Toetsingscommissie instellingen Geestelijke Gezondheidszorg; METiGG) assessed the study protocol and judged that the study could be conducted without the committee’s approval. The trial was registered at the Dutch Trial register (NTR trial number: 10340).

### Setting and Participants

The study was carried out in a Dutch mental health institution (Friesland Mental Health Care Service, city of Leeuwarden) with a catchment area of approximately 650,000 inhabitants. Data were collected from June 2011 to July 2012. The trial was completed when all patients provided their last measurement. Patients were recruited from 2 outpatient teams for psychosis: the early intervention for psychosis team (a multidisciplinary team for the treatment of patients with a first episode of psychosis) and a rehabilitation team (a multidisciplinary team for patients with chronic schizophrenia). We used broad inclusion criteria. Participants had to meet criteria for a nonaffective psychosis (brief psychotic disorder, schizophreniform disorder, schizoaffective disorder, schizophrenia, or psychotic disorder not otherwise specified) as defined by the *Diagnostic and Statistical Manual of Mental Disorders* (Fourth Edition, Text Revision) (*DSM-IV-TR*), be between age 21 and 65 years, and be fluent in Dutch. Participating professionals were all clinicians involved in the care for those patients describe previously (psychiatrists, community psychiatric nurses, psychologists). Internet or computer literacy was not part of the inclusion criteria.

To calculate the sample size, we used the SPSS SamplePower software program (IBM Corp, Armonk, NY, USA). Given an alpha of .05, a power of .80, and an effect size of .50 (based on results of a comparable study [3]), we needed n=64 per group. Because we expected a considerable amount of dropout (50%) and we wanted to investigate what proportion of patients in the participating teams would use the Web-based decision aid, we decided to include all eligible patients treated by the participating teams.

### Study Design

We conducted an open-label, 2-group, parallel, randomized controlled trial with approximately the same number of patients in each group. Patients were allocated to either an intervention group that was offered a Web-based tool to support shared decision making or a control group that received care as usual. Randomization of patients was conducted by using the online Research Randomizer [14]. We used block randomization in blocks of 8 (numbers 1 to 4 were considered intervention condition; 5 to 8 control condition). A research assistant located at the mental health institution participating in the study created a spreadsheet file listing all participants in ascending order by research number. Another research assistant located at our research center added the randomization conditions to the spreadsheet, assigning participants to the interventions.

### Treatment Conditions

#### Control Condition

Patients in the control condition received care as usual, as described in the local disease management program for the treatment of people with psychosis. Treatment modules were initially chosen by a clinician in accordance with a treatment path that a patient entered based on the staging of the disorder (first episode or stabilizing/rehabilitation phase), clinician-rated scores on the Health of the Nation Outcome Scale (HoNOS), and patient-rated scores on the Camberwell Assessment of Need Short Appraisal Schedule (CANSAS-P). During a treatment plan meeting, clinicians informed patients about the indicated treatment modules and also discussed alternatives. A final decision was made in a process of shared decision making (which was not further specified in the disease management program).

#### Intervention Condition

**Figure 1:**
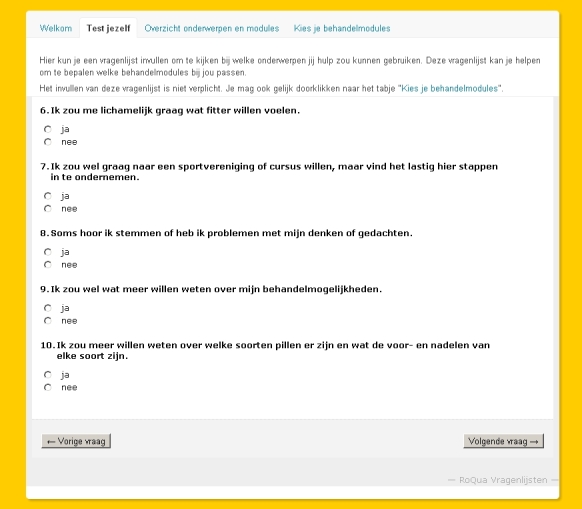
Screenshot of the first webpage with a questionnaire (in Dutch) about care needs.

**Figure 2:**
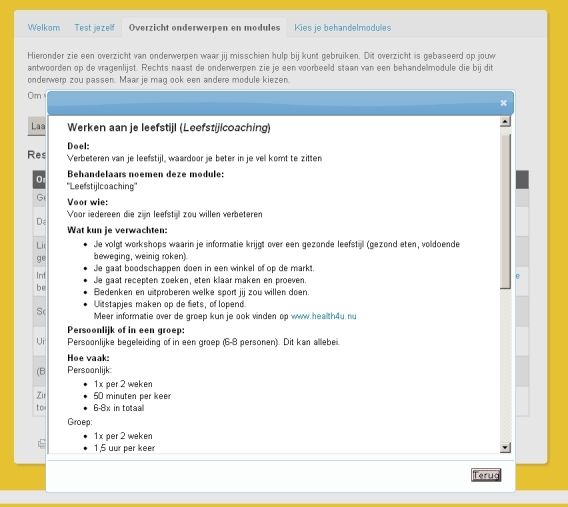
Screenshot of the second webpage including a digital catalog with descriptions of treatment modules.

Patients in the intervention condition received care as described in the local disease management program for the treatment of people with psychosis plus they were offered the opportunity to make use of the Web-based information and decision tool (see Multimedia Appendix 1). This tool is meant to support patients in acquiring an overview of their care needs and of the treatment modules provided by their mental health care organization. The tool functions as a website consisting of 3 webpages and a home page. The home page briefly explains the aim and procedure of the website. The first webpage presents a questionnaire about care needs based on items of the CANSAS-P (see Figure 1). The second webpage offers a digital catalog with descriptions of treatment modules dynamically linked to the outcomes of the questionnaire in the first webpage (see Figure 2). For instance, a reported need for more information about symptoms and medication use was linked to information in a module about psychoeducation, whereas a reported need on items about living a meaningful life and doubts about the future was linked to a module about loss and longing.

**Figure 3:**
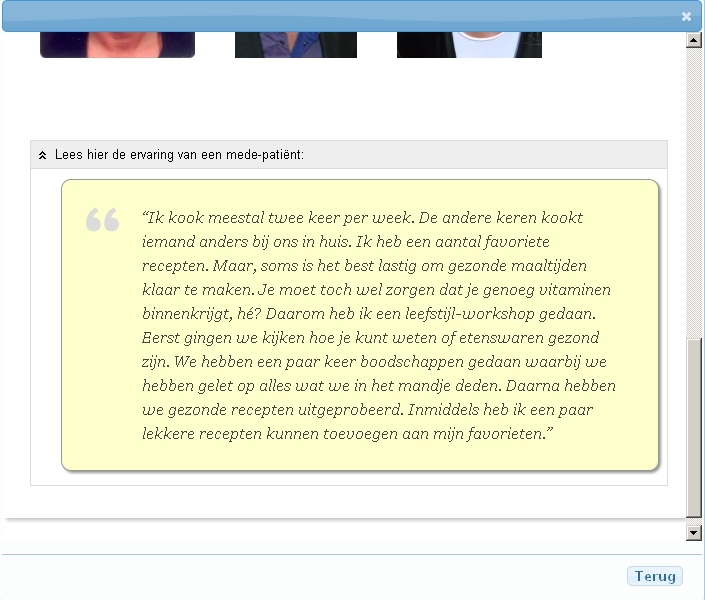
Screenshot of the second webpage with a short patient story.

In addition to this selection of modules, patients also had the opportunity to view all available treatment modules irrespective of the questionnaire outcomes. The information about the available modules in the catalog included an overview of its content and duration; a description of problems/symptoms the treatment module is usually indicated for; names, functions, and pictures of clinicians involved; a short story by a patient who tells his/her experience with the treatment module (see Figure 3); and, if available, a brief interview with a clinician who tells about his/her experience with the treatment module (advantages, disadvantages, motivation to provide the treatment, etc). The third webpage presents a list of all treatment modules in a checkbox format. The content and design of this Web-based tool was based on an earlier usability study and needs assessment [15]. During the development process, the content of the tool was validated by clinicians and patients. This content was frozen during the trial.

Patients using the Web-based tool were asked to look through the treatment modules and choose the modules of their preference by ticking the appropriate checkboxes. Patients could print the checkbox form and take it with them to their treatment plan evaluation session to discuss with their clinician.

Patients were informed about the Web-based decision aid by research nurses during a biyearly appointment for Routine Outcome Monitoring (ROM), and they were offered an information brochure. Patients were given the opportunity to use the decision aid either on their own (at their home computer, or at a computer of the service) or with support of an assistant. Furthermore, an assistant was available by phone for help on 3 days each week. Patients received a log-in account by email or on paper from an assistant. No further instructions were given about the optimal timing of frequency regarding the use of the decision aid.

### Procedure

After randomization, baseline measurement took place during a biyearly face-to-face ROM session for all participating patients. Participating clinicians were asked to complete an attitude questionnaire around the same time. Up to 6 weeks after the ROM session, patients in the intervention condition had the opportunity to make use of the Web-based tool. Approximately 6 weeks after ROM, a meeting was planned between the patient and a key clinician in which ROM results were evaluated and a new treatment plan was created or an existing one was adjusted. Patients were sent a final questionnaire by mail. Upon returning the questionnaire to our research center, they received a gift certificate worth €7.50. We deviated from the procedure described in the original research protocol in 1 important aspect: we conducted 1 follow-up measurement instead of 2 because a second follow-up meeting appeared to be not feasible within the time limits.

### Measures

#### Baseline

Self-reported quality of life was measured with the Manchester Short Assessment of Quality of Life (MANSA) [16]. Patients rate their satisfaction with life on different life domains, in 16 items on a 7-point Likert scale, ranging from very dissatisfied to very satisfied. Higher scores indicate a better quality of life.

Psychosocial functioning was measured with the HoNOS [17]. Clinicians rate patients on 12 domains on a 5-point severity scale ranging from no problem to severe or very severe problem. Lower scores indicate a better psychosocial functioning.

Symptom severity was measured with the Positive and Negative Syndrome Scale (PANSS) [18]. Clinicians rate patients during an interview on 7 items about positive symptoms, 7 items about negative symptoms, and 16 items about general psychopathology on a 7-point Likert scale ranging from absent to extreme. Lower scores indicate less symptom severity.

Patients’ preference to participate in medical decision making was measured by the decision-making preference subscale of the Autonomy Preference Index (API) [19]. Patients rate their preference on a 6-item scale in which item scores range from completely disagree (score 0) to completely agree (score 100). A higher score indicates more preference for autonomy.

#### Outcome

The primary outcome measure was patient-perceived involvement in medical decisions measured with the patient-rated Combined Outcome Measure for Risk Communication and Treatment Decision-making Effectiveness (COMRADE) [20]. The COMRADE consists of 2 subscales, satisfaction with communication and confidence in decision, comprising 20 items in total and scored on a 5-point scale. Higher scores indicate higher perceived involvement.

We used the patient-rated Client Satisfaction Questionnaire (CSQ) [21] as a secondary outcome measurement. The CSQ used in this study consists of 9 items, scored on a 4-point scale. Higher scores indicate higher satisfaction. For the intervention group, we added 6 questions about satisfaction with the Web-based decision tool.

### Analysis

Descriptive statistics were used to investigate client characteristics. Baseline measures of both conditions were compared using unpaired *t* tests or chi-square tests. Difference between the intervention and the control condition on the primary outcome measure was examined using a general linear model with adjustments for patient age and partner status (having a partner yes/no).

### Process Evaluation

The intervention described previously can be considered a complex intervention because it consists of several components (use of new technology, implementation in regular care, evaluation) and is highly dependent on the context in which it is delivered. Complex interventions are interventions that contain various interacting components of which the whole is more than the sum of its parts [22,23]. For these interventions, a randomized controlled trial needs to be supplemented by a process evaluation to evaluate their effect. Process evaluations explore implementation issues and contextual factors within the trial. They help to distinguish between ineffective interventions (failure of intervention) and badly delivered interventions (implementation failure) [22].

The process evaluation of this study consisted of

1. Open interviews with a sample of 15 patients who did and did not receive the allocated intervention. An interview guide was created in accordance with the guidelines provided by Hennink et al [24]. A verbatim transcript was created for each interview. Coding and analysis was performed with the ATLAS.ti software package.
2. Researcher observation of clinicians discussing implementation of the intervention during clinical meetings, which were recorded in a notebook by a research assistant. Themes of interest were identified by the research team and further discussed with the clinical teams when necessary.
3. A questionnaire-based survey among clinicians consisting of 3 parts: (1) investigating their attitude toward shared decision making and the use of a Web-based decision aid (based on Punter [25] and Holmes-Rovner et al [26] with internal consistency alpha =.85); (2) examining potential hampering factors for shared decision making (based on Charles et al [27]); and (3) exploring to what extent clinicians considered patients to be capable and interested in shared decision making (based on Hamann et al [3]).

This process evaluation provided data to shed light on how well the intervention was implemented, to what extent the trial outcomes were related to the quality of the implementation and the setting in which it was implemented, and what processes might have mediated these relations.

## Results

### Process Evaluation

In the process evaluation, we collected data to answer 5 questions about potential problems related to implementation and context.

The first question was: Could the outcomes be affected by a negative attitude of clinicians toward shared decision making or the Web-based decision aid? In a questionnaire-based survey, clinicians’ attitudes were investigated. On a 5-point Likert scale ranging from completely disagree to completely agree, clinicians agreed or completely agreed with 4 statements about shared decision making in general, and 9 statements about the use of a decision aid in decision-making processes. The mean total score on this scale was 3.52 (SD 0.49), meaning that most clinicians showed a positive attitude toward shared decision making and the use of decision aids. Table 1 shows to what extent clinicians agreed or disagreed with the statements.

The second question was: Do clinicians think there are too many hampering factors to realize a process of shared decision making? In addition, 18 clinicians reported that in processes of shared decision making, the following factors were often or almost always experienced as hampering decision making: patients receive contradictory advice from multiple clinicians (12/18, 67%), patients have difficulty accepting their diagnosis (12/18, 67%), and patients are indecisive (10/17, 59%). The following factors were reported as never or sometimes hampering: patients want to participate to a greater degree than the clinician prefers (15/18, 83%), patients have other interfering health problems (15/18, 83%), lack of time (14/18, 78%), cultural differences (14/18, 78%), patients bring in too much information to discuss (13/18, 72%), patients ask for a treatment that is not evidence-based (12/17, 71%), clinician has too little information to make a decision (12/17, 71%), patients do not understand the information (12/18, 67%), patients are too anxious or worried to listen to what the clinician has to say to them (11/18, 61%), and patients refuse treatment that could benefit them (10/18, 56%).

The third question was: Could the outcomes be affected by the clinicians’ judgment about patients’ capabilities and interests? Clinicians were asked to what extent they considered patients to be capable and interested in shared decision making. Of the 128 patient observations, clinicians rated most patients as being able to understand the arguments presented, being capable of making reasonable decisions, and being interested in the topics discussed as well as in participating in medical decision making. Patients who were rated by their clinicians as not capable of making decisions (score 1-3) had a significantly lower score than patients rated as capable of making decisions on both subscales of the COMRADE (COMRADE satisfaction with communication: *t*_48_=–3.857, *P*<.001; COMRADE confidence in decision: *t*_47_=–2.368, *P*=.02. This means that patients who perceived their involvement in medical decision making to be low were judged by clinicians to be less capable of participating in decision making.

The fourth question was: Could any problems be observed with fulfillment of the study protocol? Through researcher observation, several recurring themes were identified during clinical meetings in which the trial was discussed. Case managers sometimes were hesitant and felt troubled to invite intervention patients to make use of the decision tool. First, they were doubtful whether patients were able to handle either the computer program or participation in a research trial. Second, they were not sure that patients would benefit from the decision aid because not all treatment options included in the decision aid were actually offered by their organization (eg, music therapy was listed among the treatment options, but no music therapy was currently offered because of absence of a music therapist). In addition, various clinicians reported that they were unsure when to discuss outcomes of the decision aid with their patients because not all conducted a formal treatment evaluation session with their patients following their ROM assessment. Some only discussed ROM results within the clinical team and not directly with patients.

The fifth question was: Did patients experience any problems with the intervention that was not covered in the satisfaction questionnaire? Open interviews among patients who chose to use or not use the website provided some additional details on the process. First, all patients were initially informed about the decision aid by an information booklet and in a meeting with a research nurse, but most of them received additional explanation from their case manager. Some framed the decision aid predominantly within a research context (“by using the decision aid, you contribute to research”), whereas others described it as an attempt to improve services (“using the decision aid might help you reflect on the treatment you want”). This might have affected patients’ expectations of the intervention. Moreover, interviews revealed discrepancies between the policy of the local disease management program and patients’ experiences in clinical practice. Most of the interviewed patients could not remember their ROM results being discussed with them and some could not remember whether a treatment plan was created.

### Allocation and Reception of Intervention

**Figure 4:**
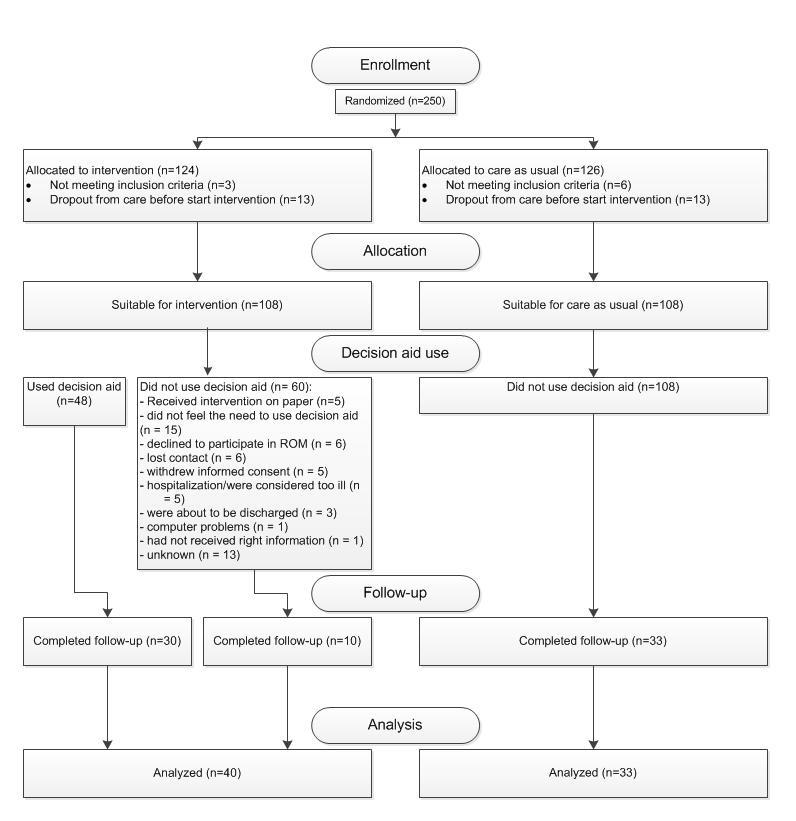
Participant flow diagram.

A total of 250 patients (n=124 intervention vs n=126 control) were included in the trial of whom 73 completed the follow-up measurement and were included in the final analysis (response rate 29.2%). Of these 73 patients, 40 were in the intervention and 33 in the control condition. Of the 40 patients in the intervention condition who completed the follow-up measurement, 30 used the decision aid. A detailed overview of the flow of participants is presented in Figure 4.

**Table 1:** Percentage of clinicians (completely) agreeing with statements about shared decision making and decision aids (n=19).

Item	Agree or completely agree, n (%)
A decision aid will cause patients to ask more questions than they would otherwise have asked	16 (84)
A decision aid will cause patients to be more involved in decision making about treatment^a^	15 (83)
All eligible patients should be invited to use the decision aid	15 (79)
Knowing risks and benefits, most patients want to decide how acceptable treatment is to them	13 (68)
Patients using a decision aid will be much better informed	13 (68)
Patients should see a decision aid before a treatment decision is made	12 (63)
Patients usually want to be an equal partner with physicians in making important treatment decisions	10 (53)
With a decision aid, I will be able to reduce time spent educating patients about treatment^a^	7 (39)
Most patients prefer the clinicians to take responsibility for their medical problems	4 (21)
Using a decision aid will reduce the risk of malpractice	4 (21)
A decision aid will eliminate the need for third-party utilization as second opinion	3 (16)
A decision aid may cause some patients to make the wrong choice	3 (16)
The majority of patients do not wish to be involved in decision making about their treatment	1 (5)

^a^n=18.

### Demographic Variables and Baseline Data

Demographic variables and baseline data of patients included in the analysis are presented in Table 2. Patients in the 2 conditions did not differ in age, Global Assessment of Functioning (GAF), MANSA, HoNOS, PANSS, API, level of education, whether they had a job or were studying, and whether or not they used antipsychotics. However, in the intervention group were fewer females (*P*=.01) and fewer patients with a partner (*P*=.01).

The patients who dropped out of the study and did not complete the follow-up measurement were slightly younger (*t*_213_=–2.129, *P*=.03) and were more often men (χ^2^_1_=5.6, *P*=.02) than the patients who did complete the outcome measurement. They did not differ on any of the other baseline characteristics. Patients in the intervention condition who received the allocated intervention versus those who did not receive the intervention did not differ on all baseline characteristics.

**Table 2:** Demographic variables and baseline data of study participants.

Variable		Intervention (n=40)	Control (n=33)	*P* ^a^
Age (years), mean (SD)		37 (12.35)	40 (13.47)	.35
Sex (female), n (%)		13 (33)	21 (64)	.01
Education (≥ 12 years), n		10 (n=12)	10 (n=12)	.99
Job or study, n (%)		13 (33; n=39)	16 (48)	.23
Partner, n (%)		9 (23; n=39)	18 (55)	.01
Use of antipsychotics, n (%)		29 (73)	22 (67)	.60
**Test, mean (SD)** ^b^				
	GAF	61.8 (9.08)	57.4 (10.91)	.06
	MANSA	60.7 (9.50)	62.3 (13.26)	.58
	HoNOS	7.7 (4.75)	8.4 (4.32)	.53
	PANSS total score	13.3 (5.24)	15.4 (5.51)	.13
	API	55.7 (12.72)	52.7 (12.96)	.38
Number of patients from the first episode of psychosis team within condition, n (%)		16 (40)	13 (39)	.99

^a^Using Fisher exact test or *t* test.

^b^GAF: Global Assessment of Functioning; MANSA: Manchester Short Assessment of Quality of Life; HoNOS: Health of the Nation Outcome Scales; PANSS: Positive and Negative Syndrome Scale; API: Autonomy Preference Index.

### Patient Involvement in Treatment Planning and Their Satisfaction With Care

Intention-to-treat analyses showed that patients in the intervention condition did not differ from patients in the control condition in their perceived involvement in medical decision making (COMRADE) after they had used the Web-based decision aid (COMRADE satisfaction with communication: *F*_1,68_=0.422, *P*=.52; COMRADE confidence in decision: *F*_1,67_=0.086, *P*=.77; see also Table 3). This was the primary outcome measure. Patients also did not differ in self-reported satisfaction with care (CSQ) (*F*_1,70_=0.014, *P*=.91).

Per protocol analyses also showed that patients in the intervention condition who received the allocated intervention and completed the follow-up measure (n=30) did not differ regarding their perceived involvement in medical decision making and in satisfaction with care from patients in the control condition (n=33) (COMRADE satisfaction with communication: *F*_1,57_=0.155, *P*=.70; COMRADE confidence in decision: *F*_1,56_=0.413, *P*=.52; CSQ: *F*_1,60_=0.789, *P*=.34).

In an additional analysis, patients in the intervention condition who received the allocated intervention (n=30) were compared to patients in the intervention condition who did not receive the allocated intervention (n=10). No differences were found for patients’ perceived involvement in medical decision making (COMRADE satisfaction with communication: *F*_1,36_=0.642, *P*=.43; COMRADE confidence in decision: *F*_1,36_=2.310, *P*=.14). Patients did, however, differ on the secondary outcome self-reported satisfaction with care (*F*_1,37_=6.306, *P*=.02). Patients who received the allocated intervention were less satisfied than patients who did not.

**Table 3:** Primary outcome data of patients’ perceived involvement in medical decision making at the end of the study using the Combined Outcome Measure for Risk Communication and Treatment Decision-making Effectiveness (COMRADE) test.

COMRADE subscale^a^	Intervention, mean (SD)	Control, mean (SD)	*F* (df)	*P*
Satisfaction with communication (n=73)	38.25 (1.06	37.19 (1.165)	0.422 (1,68)	.52
Confidence in decision (n=70)	38.78 (1.17)	38.72 (1.307)	0.086 (1,67)	.77

^a^Group differences were analyzed using a general linear model with age and partner status as covariates.

### Use of and Satisfaction With the Web-Based Decision Aid

Of the 48 patients who used the Web-based decision aid, 12 used their own computer, 12 used the computer at the clinic, and 6 used a computer elsewhere. Furthermore, 13 used the decision aid independently, 16 received assistance from a professional (often their case manager), and 1 received assistance from someone else. First-episode patients used their own computer and used the decision aid without assistance more often than chronic patients did. Of the 48 patients who used the website, 34 (71%) used full functionality of the Web-based decision aid, meaning that patients completed the care needs assessment (first webpage of the website) and looked through the digital catalog with descriptions of treatment modules (second webpage of the website). More than half of them were long-term care patients (27/48, 56%).

In the intervention condition, 29 of 48 patients who used the decision aid (60%) completed questions about their satisfaction with the decision aid. They agreed or completely agreed with the following statements: “I have been well informed about the treatment options offered by Friesland Mental Health Care Service by the decision aid” (22/29, 76%), “The advice presented by the decision aid has helped me to reflect on what I want” (22/29, 76%), “The decision aid was easy to use” (20/28, 71%), “I would recommend the decision aid to others” (20/27, 74%) and “The decision aid helped me to get a clearer view on what my problem areas or points of interest are” (17/28, 61%). Patients were divided on whether the decision aid helped them to better prepare the evaluation meeting with their clinicians, 44% (12/27) said it did help; 56% (15/27) were neutral or said it did not help. Means and standard deviations can be found in Table 4.

**Table 4:** Secondary outcome data of patients’ satisfaction with the Web-based decision aid.

Question	Mean (SD)^a^
I have been well informed about the treatment options offered by the GGZ Friesland by the decision aid (n=29)	3.93 (0.84)
The advice presented by the decision aid has helped me to reflect on what I want (n=29)	3.86 (0.79)
As a consequence of using the decision aid, I was better prepared for the evaluation meeting with my clinician (n=27)	3.33 (0.78)
The decision aid helped me to get a clearer view on what my problem areas or points of interest are (n=28)	3.61 (0.92)
The decision aid was easy to use (n=28)	3.79 (1.07)
I would recommend the decision aid to others (n=27)	3.89 (0.75)

^a^Scores ranged from 0 (completely disagree) to 5 (completely agree).

## Discussion

### Principal Findings

In this study, we report on a clinical trial and process evaluation of a Web-based intervention to facilitate shared decision making for people with psychotic disorders.

To be able to explore potential implementation issues and contextual problems within the trial, we conducted a process evaluation. This evaluation showed that no significant problems could be observed in the attitude and beliefs of clinicians. Participating clinicians had an overall positive attitude toward shared decision making. They reported that their patients were generally interested in and capable of participating in medical decision making, they considered patient decision aids be to potentially helpful, and they judged relatively few factors to be hampering in a shared decision-making process. However, problems were observed in the implementation of the intervention. Not all patients in the intervention group were actually offered the possibility to use the decision aid and, more importantly, ROM and treatment evaluation meetings in which the treatment plan was to be discussed in a process of shared decision making did not always take place. Moreover, interviews indicate that the Web-based intervention might have been framed differently to different patients, which may have shaped their expectations and affected their evaluation. An interesting finding in the process evaluation was that patients who perceived their involvement in medical decision making as low were judged by clinicians to be less capable of participating in decision making. This could imply that patients participate less because they are less capable. Nevertheless, we cannot rule out that patients participate less because clinicians consider them less capable and, therefore, provide less opportunities for patients to participate in decision making.

The findings of our trial show that more than one-third of the patients who were provided access to the Web-based decision aid chose to use it and most used full functionality of the decision aid whether they were first-episode patients or long-term patients. Users and nonusers did not differ in demographic variables. At least one-quarter of the patients used their own computer and a similar proportion used the decision aid without assistance. Most of these were first-episode patients. On average, users of the decision aid reported to be rather satisfied with the system. Nevertheless, primary outcome results could not support the assumption that the use of electronic decision aids increases patient involvement in medical decision making, neither in intention-to-treat analyses nor in per protocol analyses. In addition, we did not find a difference in self-reported satisfaction with care between patients who had the opportunity to use the decision aid versus those who did not.

Our outcomes are in-line with the study by Woltmann et al [12] who found no difference in patient satisfaction between intervention and control group. However, they contradict the findings by Hamann et al [3] and Steinwachs et al [13] who found a positive effect of decision aids on patients’ involvement in consultations with their clinicians. This discrepancy can be explained by several reasons. First, the decision aids used in these trials differed in format (Hamann et al [3] used a printed decision aid) and content. Some decision aids primarily concentrated on pharmacological information, whereas others had a broader focus. Second, settings were different. In our study, patients could use the decision aid either in the clinic or at home, with or without assistance, whereas in the trial by Hamann et al [3], patients used the decision aid in a psychiatric ward with assistance of trained nurses. The setting in the study by Steinwachs et al [13] was not described. Third, our response rate was very low. This is partly because of the naturalistic setting of our study. However, response rates are highly dependent on selection criteria used in studies. For example, if Steinwachs et al [13] included all eligible patients (eg, not excluding patients who were considered unsuitable by their clinician), their response rate would have been comparable. Fourth, the outcome measures used in our study might have been too unspecific, indirect, or insensitive to detect differences in a small sample. The COMRADE measures patients’ perceived involvement in medical decision making with a self-report questionnaire that is completed retrospectively. What actually happens during the conversation between patient and clinician remains a black box. Furthermore, research has shown that ratings on patient satisfaction questionnaires tend to be more optimistic than patients’ actual evaluations [28,29], implying that there may be less differentiation in the response behavior. Finally, discrepancies could, but are not likely to, be explained by lack of need for shared decision making in our patient sample. Patients’ mean score on the API, which indicates their preference for participation in medical decision making, was comparable to or even slightly higher than previous studies in people with schizophrenia [2,3,30].

### Strengths and Limitations

Given the problems observed in the process evaluation, the intervention designed for our study appeared not to fit in optimally with the routine practice of the participating clinical care teams. Therefore, the lack of significant effects on our outcome measures cannot be solely attributed to failures intrinsic to the intervention. Future studies might benefit from a stronger integration of shared decision-making interventions in clinical practice by training clinical teams in using (output) from decision aids. A comprehensive overview of the working flow of patients and clinicians is crucial to realize this integration. Given the low response rate and moderate participation rate in this study, it may also be desirable to investigate efficacy of decision aids in a less naturalistic setting in which participating patients are selected more strictly and required to use the decision aid before performing a naturalistic study. In addition, special attention should be paid to the selection of outcome measures used to assess the shared decision-making process. Instruments focusing on satisfaction might suffer from ceiling effects, and instruments such as the COMRADE may be too broad and indirect to detect changes in the decision-making process. A better alternative is to record conversations between clinicians and patients and observe what is actually happening within that conversation. A promising instrument for this may be the recently developed Mappin’SDM [31], which combines patient, clinician, and observer perspectives. It is also important to note that using Web-based decision aids or support systems does not need to be a desirable target for all patients. Although some may benefit from new tools, others might not. It would be most helpful to know what works for whom.

The main limitation of this study is the weak implementation of the study protocol; as a result, it is difficult to draw firm conclusions about the study’s outcomes. We tried to prevent this by preparing the participating teams before the start of the trial and keeping closely in touch during the trial (eg, being present at clinical meetings, functioning as helpdesk, sending individual emails to participating clinicians as reminders of specific actions). Another important limitation is the large numbers of dropouts before the follow-up measurement, even though patients were offered a small gift for returning their completed questionnaire.

Our study also has strengths. Most importantly, it affirms previous findings that many people with a severe mental illness can work with electronic decision aids, either with or without assistance, at the clinic or at home. Furthermore, our study provides insight in variation among the population concerning interest in and use of electronic decision aids. Our results suggest that part of the population is not able or does not feel the need to work with these decision aids. Based on our results, the ratio of users versus nonusers could be 50-50. Another strength is that we collected detailed information about allocation and reception of the intervention with varying illness durations, and we included a process evaluation that allowed us to perform a critical analysis on the trial results.

### Conclusion

The development of electronic decision aids to facilitate shared medical decision making is encouraged and many people with a psychotic disorder can work with them. This holds for both first-episode patients and long-term care patients, although the latter group might need more assistance. However, effects of decision aids on patient participation in medical decision making have not been consistently demonstrated.

## Multimedia Appendix 1

Video of the Web-based decision aid.

## Multimedia Appendix 2

CONSORT-EHEALTH Checklist V1.6.2 [32].

## Abbreviations

API: Autonomy Preference Index
CANSAS-P: Camberwell Assessment of Need Short Appraisal Schedule–Self-Report Version
COMRADE: Combined Outcome Measure for Risk Communication and Treatment Decision-making Effectiveness
CSQ: Client Satisfaction Questionnaire
DSM-IV-TR: Diagnostic and Statistical Manual of Mental Disorders (Fourth Edition, Text Revision)
GAF: Global Assessment of Functioning
HoNOS: Health of the Nation Outcome Scales
MANSA: Manchester Short Assessment of Quality of Life
METiGG: Medisch-ethische Toetsingscommissie instellingen Geestelijke Gezondheidszorg (Dutch medical ethical committee for mental health care)
NTR: NederlandsTrial Register (Dutch trial register)
PANSS: Positive and Negative Syndrome Scale
ROM: Routine Outcome Monitoring
