# Supplementary material for: A Web-Based Tool to Support Shared Decision Making for People With a Psychotic Disorder: Randomized Controlled Trial and Process Evaluation
Source: J Med Internet Res. 2013 Oct 7;15(10):e216. doi: 10.2196/jmir.2851 (PMC3806550; doi:10.2196/jmir.2851)
Supplement: Supplementary file 2 [file jmir_v15i10e216_app2.pdf]

CONSORT-EHEALTH Checklist V1.6.2 Report

(based on CONSORT-EHEALTH V1.6), available at [http://tinyurl.com/consort-ehealth-v1-6].

2851

Date completed

7/26/2013 5:54:44

by

Lian van der Krieke

A web-based tool to support shared decision-making for people with a psychotic disorder – a randomized clinical trial and process evaluation

TITLE

1a-i) Identify the mode of delivery in the title

"A web-based tool to ... "

1a-ii) Non-web-based components or important co-interventions in title

Not applicable. We do not use co-interventions.

1a-iii) Primary condition or target group in the title

"...for people with a psychotic disorder ..."

ABSTRACT

1b-i) Key features/functionalities/components of the intervention and comparator in the METHODS section of the ABSTRACT

"Patients in the intervention condition (n=124) were provided an account to access a web-based information and decision tool aimed to support patients in acquiring an overview of their needs and appropriate treatment options provided by their mental health care organization."

1b-ii) Level of human involvement in the METHODS section of the ABSTRACT

"Patients were given the opportunity to use the web-based tool either on their own, at their home computer or at one of the computers of the service, or with support of an assistant. "

1b-iii) Open vs. closed, web-based (self-assessment) vs. face-to-face assessments in the METHODS section of the ABSTRACT

"Patients in the intervention condition (n=124) were provided an account to access a web-based information and decision tool ..."

1b-iv) RESULTS section in abstract must contain use data

"Seventy-three patients completed the follow-up measurement and were included in the final analysis (response rate 29%). Results show that almost half of the patients who were provided access to the web-based decision aid did use it, and the majority of them used its full functionality. However, no differences were found between intervention and control condition on perceived involvement in medical decision-making (COMRADE risk communication $F(1) = 0.422$, $p = .518$; COMRADE confidence in decision $F(1)= 0.086$; $p = 0.770$). In addition, results of the process evaluation suggest that the intervention did not optimally fit in with routine practice of the participating teams. "

1b-v) CONCLUSIONS/DISCUSSION in abstract for negative trials

"However, results of this paper could not support the assumption that the use of electronic decision aids increases patient involvement in medical decision-making. This can be partly explained by a weak implementation of the study protocol and a low response rate. "

INTRODUCTION

2a-i) Problem and the type of system/solution

"This paper reports on a randomized clinical trial and process evaluation of a web-based intervention to facilitate shared decision-making, with or without assistance, in people with psychotic disorders. "

2a-ii) Scientific background, rationale: What is known about the (type of) system

"Shared decision-making in mental health care has been dubbed an ethical imperative [1]. Since the rise of recovery-oriented medicine, patients have been acknowledged as experiential experts and equal partners in communication with clinicians. Research has shown that people with severe and persistent mental disorders are no exception. People with psychotic disorders are able and willing to participate in medical decision-making [2, 3]. However, the desire for participation is greater than the amount of participation they actually experience [4, 5]. A range of obstacles are hampering successful implementation. Most clinicians believe in the benefits of shared decision-making, but time constraints and a large number of clinical responsibilities retain them from practicing it [6, 7]. Moreover, patients may not be used to actively participate in medical decision-making and they sometimes lack access to medical information that is easily intelligible [8].

Therefore, Drake and Deegan [9] have stressed the need for decision aids and support centers to ensure the development of an infrastructure that facilitates the practice of shared decision-making. Several initiatives have been developed in this area. For instance, in Germany, Hamann et al. [3] investigated the effectiveness of a shared decision-making intervention with a printed decision aid for inpatients with schizophrenia. They found that patients using the decision aid, compared to a control group that received care as usual, had better knowledge about their disease and had a higher perceived involvement in medical decisions [3]. Recently, a special case was made for electronic decision aids [10], as they have various advantages over paper-based decision aids, such as presenting personalized information based on smart algorithms. So far, three electronic decision aids have been developed and investigated to support shared decision-making in the treatment planning for people with severe mental disorders, but the results are inconsistent [11, 12]. A pilot study by Deegan et al. [11] showed that outpatients were able to work with a web-based program to support shared decision-making on psychopharmacological consultation. Patients used the program on computers at the clinic where experiential experts were available for assistance. Two small-scale randomized clinical trials were conducted [12, 13]. The first trial showed that patients were able to electronically make up their own care plan, but there was no difference between intervention and control group on satisfaction with the care planning process, which was the primary outcome [12]. The second trial reported that a web-based support system encouraging patients to discuss their current status and treatment with their clinician resulted into patients being more verbally active during health visits [13].

More evidence is needed to determine whether electronic decision aids are helpful in clinical practice, and lead to increased patient involvement and better outcomes. In addition, more information is needed on what proportion of patients is willing and able to work with web-based decision aids, and in what form (with or without assistance, using their own computer or one at the clinic)."

METHODS

3a) CONSORT: Description of trial design (such as parallel, factorial) including allocation ratio

"Our aim was to investigate this intervention in a naturalistic setting, meaning that all eligible patients were included in order to be able to determine how many of them would actually use the decision aid. "

3b) CONSORT: Important changes to methods after trial commencement (such as eligibility criteria), with reasons

"We deviated from the procedure described in the original research protocol in one important aspect: we conducted one follow-up measurement instead of two, as a second follow-up meeting appeared not to be feasible within time limits."

3b-i) Bug fixes, Downtimes, Content Changes

Not applicable. The web-based decision aid was 'frozen' during the trial.

4a) CONSORT: Eligibility criteria for participants

"We used broad inclusion criteria. Participants had to meet DSM-IV-TR criteria for a non-affective psychosis (brief psychotic disorder, schizophreniform disorder, schizo-affective disorder, schizophrenia or psychotic disorder not otherwise specified), be aged between 21 and 65 years old, and be fluent in Dutch. Participating professionals were all clinicians involved in the care for those patients describe above (psychiatrists, community psychiatric nurses, psychologists). "

4a-i) Computer / Internet literacy

"Internet or computer literacy was not part of the inclusion criteria."

4a-ii) Open vs. closed, web-based vs. face-to-face assessments:

"The study was carried out in a Dutch mental health institution (GGZ Friesland, city of Leeuwarden) with a catchment area of approximately 650.000 inhabitants. Data was collected from June 2011 to July 2012. The trial was completed when all patients received their last measurement. Patients were recruited from two outpatient teams for psychosis: the early intervention for psychosis team (a multidisciplinary team for the treatment of patients with a first episode of psychosis) and a rehabilitation team (a multidisciplinary team for patients with chronic schizophrenia). "

4a-iii) Information giving during recruitment

"Patients were informed about the web-based decision aid by research nurses, during a bi-yearly appointment for routine outcome monitoring (ROM), and they were offered an information brochure. "

"Informed consent was obtained by research nurses. Patients were provided with an information brochure and they received a phone number and email address of a research assistant who they could contact for further information. A few weeks after the initial information, patients were asked whether they were willing to participated in the trial. "

4b) CONSORT: Settings and locations where the data were collected

"The study was carried out in a Dutch mental health institution (GGZ Friesland, city of Leeuwarden) with a catchment area of approximately 650.000 inhabitants. Data was collected from June 2011 to July 2012. The trial was completed when all patients received their last measurement. Patients were recruited from two outpatient teams for psychosis: the early intervention for psychosis team (a multidisciplinary team for the treatment of patients with a first episode of psychosis) and a rehabilitation team (a multidisciplinary team for patients with chronic schizophrenia). "

4b-i) Report if outcomes were (self-)assessed through online questionnaires

"After randomization, baseline measurement took place during a bi-yearly face-to-face routine outcome monitoring session for all participating patients."
"Patients were sent a final questionnaire by mail. Upon returning the questionnaire to our research center, they received a gift certificate of 7.50 euros."

4b-ii) Report how institutional affiliations are displayed

We do not suspect that institutional affiliations will have affected the outcomes. The study took place at a mental health institution with patients receiving care that mental health institution.

5) CONSORT: Describe the interventions for each group with sufficient details to allow replication, including how and when they were actually administered

5-i) Mention names, credential, affiliations of the developers, sponsors, and owners

Not applicable. There is no commercial product involved.

5-ii) Describe the history/development process

"The third tab presents a list of all treatment modules in a checkbox format. The content and design of this web-based tool was based on an earlier usability study.[14] "

5-iii) Revisions and updating

"This content was 'frozen' during the trial."

5-iv) Quality assurance methods

"During the development process, the content of the tool was validated by clinicians and patients."

5-v) Ensure replicability by publishing the source code, and/or providing screenshots/screen-capture video, and/or providing flowcharts of the algorithms used

"...(see Multimedia Appendix 1)."

5-vi) Digital preservation

All pages are behind login. Therefore we created a video showing the full functionality of the application. See Multimedia Appendix 1.

5-vii) Access

"Patients received a login account by email, or on paper from an assistant."

The web-based decision aid was only availabe for patients of the Dutch mental health institution where the study was carried out. This can be taken from the context of the paper.

We did not pay patients for using the web-based decision aid, but we did provide them a gift for completing the follow-up measurement:
"Upon returning the questionnaire to our research center, they received a gift certificate of 7.50 euros"

5-viii) Mode of delivery, features/functionalities/components of the intervention and comparator, and the theoretical framework

Intervention condition:
"Patients in the intervention condition received care as described in the local disease management program for the treatment of people with psychosis plus they were offered the opportunity to make use of the web-based information and decision tool (see Multimedia Appendix 1). This tool is meant to support patients in acquiring an overview of their care needs and of the treatment modules provided by their mental health care organization. The tool functions as a website consisting of three tabs and a homepage. The homepage briefly explains the aim and procedure of the website. The first tab presents a questionnaire about care needs, based on items of the CANSAS-P. The second tab offers a digital catalogue with descriptions of treatment modules dynamically linked to the outcomes of the questionnaire in the first tab. For instance, a reported need for more information about symptoms and medication use was linked to information about the module psychoeducation, while a reported need on items about living a meaningful life and doubts about the future was linked to a module about loss and longing. In addition to this selection of modules, patients also had the opportunity to view all available treatment modules, irrespective of the questionnaire outcomes. The information about the available modules in the catalogue included an overview of its content and duration, a description of problems/symptoms the treatment module is usually indicated for, names, functions and pictures of clinicians involved, a short story by a patient who tells his/her experience with the treatment module, and, if available, a brief interview with a clinician who tells about his/her experience with the treatment module (advantages, disadvantages, motivation to provide the treatment, etc.). The third tab presents a list of all treatment modules in a checkbox format. The content and design of this web-based tool was based on an earlier usability study.[14] During the development process, the content of the tool was validated by clinicians and patients. This content was 'frozen' during the trial.
Patients using the web-based tool were asked to look through the treatment modules and to choose the modules of their preference by ticking the according checkboxes. Patients could print the checkbox form and take it with them to the treatment plan evaluation session to discuss it with their clinician.
Patients were informed about the web-based decision aid by research nurses, during a bi-yearly appointment for routine outcome monitoring (ROM), and they were offered an information brochure. Patients were given the opportunity to use the decision aid either on their own, at their home computer or at one of the computers of the service, or with support of an assistant. Furthermore, an assistant was available by phone for help for three days a week. Patients received a login account by email, or on paper from an assistant. No further instructions were given about the optimal timing of frequency regarding the use of the decision aid."

Control condition:
"Patients in the control condition received care as usual, as described in the local disease management program for the treatment of people with psychosis. Treatment modules were initially chosen by a clinician in accordance with a treatment path that a patient entered based on the staging of the disorder (first episode or stabilizing/rehabilitation phase), clinician rated scores on the HoNOS and patient rated scores on the Camberwell Assessment of Need Short Appraisal Schedule (CANSAS-P). During a treatment plan meeting, clinicians informed patients about the indicated treatment modules, and also discussed alternatives. A final decision was to be made in a process of 'shared decision-making' (which was not further specified in the disease management program). "

5-ix) Describe use parameters

"No further instructions were given about the optimal timing of frequency regarding the use of the decision aid. "

5-x) Clarify the level of human involvement

"Patients were given the opportunity to use the decision aid either on their own, at their home computer or at one of the computers of the service, or with support of an assistant. Furthermore, an assistant was available by phone for help for three days a week. "

5-xi) Report any prompts/reminders used

Not applicable.

5-xii) Describe any co-interventions (incl. training/support)

Not applicable.

6a) CONSORT: Completely defined pre-specified primary and secondary outcome measures, including how and when they were assessed

"Outcome
The primary outcome measure was patient perceived involvement in medical decisions measured with:
•The patient-rated Combined Outcome Measure for Risk Communication and Treatment Decision-making Effectiveness (COMRADE; [19]). The COMRADE consists of two sub-scales 'risk communication' and 'confidence in decision', in total comprising 20 items, scored on a 5-point scale. Higher scores indicate higher perceived involvement.
Secondary outcome measures:
•The patient-rated Client Satisfaction Questionnaire (CSQ; [20]). The CSQ used in this study consists of 9 items, scored on a 4-point scale. Higher scores indicate higher satisfaction.
•For the intervention group, we added questions about satisfaction with the web-based decision tool.
"

"After randomization, baseline measurement took place during a bi-yearly face-to-face routine outcome monitoring session for all participating patients"
"Up to six weeks after the ROM session, patients in the intervention condition had the opportunity to make use of the web-based tool. Approximately six weeks after ROM , a meeting was planned between patient and key clinician in which ROM results were evaluated, and a new treatment plan was created or an existing one adjusted. Patients were sent a final questionnaire by mail. Upon returning the questionnaire to our research center, they received a gift certificate of 7.50 euros"

6a-i) Online questionnaires: describe if they were validated for online use and apply CHERRIES items to describe how the questionnaires were designed/deployed

Not applicable.

6a-ii) Describe whether and how "use" (including intensity of use/dosage) was defined/measured/monitored

Use of the web-based decion aid was mainly measured with a questionnaire. Numbers can be found in the flow diagram.

6a-iii) Describe whether, how, and when qualitative feedback from participants was obtained

"For the intervention group, we added questions about satisfaction with the web-based decision tool."
"...open interviews with a sample of 15 patients that did and did not receive the allocated intervention, "

6b) CONSORT: Any changes to trial outcomes after the trial commenced, with reasons

Not applicable.

7a) CONSORT: How sample size was determined

7a-i) Describe whether and how expected attrition was taken into account when calculating the sample size

"To calculate the sample size, we used the Sample Power software program. Given an alpha of 0.05, a power of 0.80 and an effect size of 0.50 (based on results of a comparable study[3]), we would need n = 64 per group. Since we expected a considerable amount of dropout (50%) and we wanted to investigate what proportion of patients in the participating teams would use the web-based decision aid, we decided to include all eligible patients treated by the participating teams."

7b) CONSORT: When applicable, explanation of any interim analyses and stopping guidelines

"The trial was completed when all patients received their last measurement. "

8a) CONSORT: Method used to generate the random allocation sequence

"Randomization of patients was conducted by using the online Research Randomizer (www.randomizer.org)."
8b) CONSORT: Type of randomisation; details of any restriction (such as blocking and block size)
"We used block randomization in blocks of 8 (number 1 to 4 was considered intervention condition, 5 to 8 control condition)."
9) CONSORT: Mechanism used to implement the random allocation sequence (such as sequentially numbered containers), describing any steps taken to conceal the sequence until interventions were assigned
" A research assistant located at the mental health institution participating in the study created an excel file listing all participants in an ascending order on research number. Another research assistant, located at our research center, added the randomization conditions to the excel file, thereby assigning participants to the interventions. "
10) CONSORT: Who generated the random allocation sequence, who enrolled participants, and who assigned participants to interventions
" A research assistant located at the mental health institution participating in the study created an excel file listing all participants in an ascending order on research number. Another research assistant, located at our research center, added the randomization conditions to the excel file, thereby assigning participants to the interventions. "
11a) CONSORT: Blinding - If done, who was blinded after assignment to interventions (for example, participants, care providers, those assessing outcomes) and how
11a-i) Specify who was blinded, and who wasn't
Neither participants nor clinicians were blinded to the conditions. "We conducted an open-label two-group parallel randomized clinical trial."
11a-ii) Discuss e.g., whether participants knew which intervention was the "intervention of interest" and which one was the "comparator"
Patients knew.
11b) CONSORT: If relevant, description of the similarity of interventions
Not applicable.
12a) CONSORT: Statistical methods used to compare groups for primary and secondary outcomes
"Descriptive statistics were used to investigate client characteristics. Baseline measures of both conditions were compared using unpaired t-tests/chi2 tests. Difference between the intervention and the control condition on the primary outcome measure was examined using a general linear model with adjustments for patient age and partner status (having a partner yes/no)."
12a-i) Imputation techniques to deal with attrition / missing values
Our primary method was an intention-to-treat analysis.
12b) CONSORT: Methods for additional analyses, such as subgroup analyses and adjusted analyses
We also conducted a per-protocol analysis: "Per protocol analyses also showed that ..."
"In an additional analysis, patients in the intervention condition who received the allocated intervention (n=30) were compared to patients in the intervention condition who did not receive the allocated intervention (n=10). "

RESULTS

13a) CONSORT: For each group, the numbers of participants who were randomly assigned, received intended treatment, and were analysed for the primary outcome
See flow diagram.
13b) CONSORT: For each group, losses and exclusions after randomisation, together with reasons
See flow diagram.
Also:
"A number of 250 patients (n= 124 intervention versus n=126 control) were included in the trial of whom 73 completed the follow-up measurement and were included in the final analysis (response rate 29%). Of these 73 patients, 40 were in the intervention and 33 in the control condition. Of the 40 patients in the intervention condition who completed the follow-up measurement, 30 used the decision aid. A detailed overview of the flow is presented in Figure 1."
13b-i) Attrition diagram
Not applicable. All information is provided in 13b.
14a) CONSORT: Dates defining the periods of recruitment and follow-up
"Data was collected from June 2011 to July 2012."
14a-i) Indicate if critical "secular events" fell into the study period
Not applicable
14b) CONSORT: Why the trial ended or was stopped (early)
Not applicable.
15) CONSORT: A table showing baseline demographic and clinical characteristics for each group
See Table 1.
15-i) Report demographics associated with digital divide issues
Not available. But "Internet or computer literacy was not part of the inclusion criteria."
16a) CONSORT: For each group, number of participants (denominator) included in each analysis and whether the analysis was by original assigned groups
16-i) Report multiple "denominators" and provide definitions
We provided information about the sample size (n) in every table and in the flow diagram.
16-ii) Primary analysis should be intent-to-treat
Our primary method was intention-to-treat analysis. We also did a secondare per protocol analysis.
17a) CONSORT: For each primary and secondary outcome, results for each group, and the estimated effect size and its precision (such as 95% confidence interval)
See Table 1-5
17a-i) Presentation of process outcomes such as metrics of use and intensity of use
"Of the 48 patients that used the web-based decision aid it is known that 12 used their own computer, 12 used the computer at the clinic and 6 used a computer elsewhere. Furthermore, 13 used the decision aid independently, 16 received assistance from a professional (often their case manager) and 1 received assistance from someone else. First episode patients more often than chronic patients used their own computer and used the decision aid without assistance. Thirty-four of the 48 patients who used the website (71%) were recorded to have used full functionality of the web-based decision aid, meaning that patients completed the care needs assessment (first tab of the website) and looked through the digital catalogue with descriptions of treatment modules (second tab of the website). More than half of them were long-term care patients (56%)."
17b) CONSORT: For binary outcomes, presentation of both absolute and relative effect sizes is recommended
Not applicable.
18) CONSORT: Results of any other analyses performed, including subgroup analyses and adjusted analyses, distinguishing pre-specified from exploratory

"Per protocol analyses also showed that ..."
"In an additional analysis, patients in the intervention condition who received the allocated intervention (n=30) were compared to patients in the intervention condition who did not receive the allocated intervention (n=10). "

18-i) Subgroup analysis of comparing only users

Our primary analysis was an intention-to-treat analysis. We did add a per protocol analysis, but only secondary.

19) CONSORT: All important harms or unintended effects in each group

See below.

19-i) Include privacy breaches, technical problems

One patient experienced computer problems on his own computer. This is mentioned in the Flow diagram.

19-ii) Include qualitative feedback from participants or observations from staff/researchers

We conducted a process evaluation, including interviews and observation. Results of this evaluation are presented in the Results section.

DISCUSSION

20) CONSORT: Trial limitations, addressing sources of potential bias, imprecision, multiplicity of analyses

20-i) Typical limitations in ehealth trials

"The main limitation of this study is the weak implementation of the study protocol, as a result of which it is difficult to draw firm conclusions about the study's outcomes. We have tried to prevent this by preparing the participating teams before the start of the trial, and keeping closely in touch during the trial (e.g. being present at clinical meetings, functioning as helpdesk, sending individual emails to participating clinicians as reminders of specific actions). Another important limitation is the large amount of drop out before the follow-up measurement, even though patients were offered a small gift for returning their completed questionnaire. "

21) CONSORT: Generalisability (external validity, applicability) of the trial findings

21-i) Generalizability to other populations

We compare the results of our trial to other studies and discuss those aspects that are specific for our situation.
"This discrepancy can be explained by several reasons. First, the decision aids used in these trials differed in format (Hamann et al. [3] used a printed decision aid) and content. Some decision aid mainly concentrated on pharmacological information, while others had a broader focus. Second, settings were different. In our study, patients could use the decision aid either in the clinic or at home, with or without assistance, whereas in the trial by Hamann et al. [3] patients used the decision aid in a psychiatric ward with assistance of trained nurses"

21-ii) Discuss if there were elements in the RCT that would be different in a routine application setting

22) CONSORT: Interpretation consistent with results, balancing benefits and harms, and considering other relevant evidence

22-i) Restate study questions and summarize the answers suggested by the data, starting with primary outcomes and process outcomes (use)

"In this study we report on a clinical trial and process evaluation of a web-based intervention to facilitate shared decision-making for people with psychotic disorders. Our findings show that almost half of the patients who were provided access to the web-based decision aid chose to use it, and the majority of them used full functionality of the decision aid, whether first episode patients or long-term patients. Users and non-users did not differ in demographic variables. At least a quarter of the patients used their own computer and a similar proportion used the decision aid without assistance. Most of them were first episode patients. On average, users of the decision aid reported to be rather satisfied with the system. Nevertheless, primary outcome results could not support the assumption that the use of electronic decision aids increases patient involvement in medical decision-making, neither in intention to treat analyses nor in per protocol analyses. In addition, we did not find a difference in self-reported satisfaction with care between patients who had the opportunity to use the decision aid versus those who did not. "

22-ii) Highlight unanswered new questions, suggest future research

"...effects of decision aids on patient participation in medical decision-making have not been consistently demonstrated. "
"Future studies might benefit from a stronger integration of shared decision-making interventions in the clinical practice by training clinical teams in using (output) from decision aids. In addition, special attention should be paid to the selection of outcome measures used to assess the shared decision-making process. Instruments focusing on satisfaction might suffer from ceiling effects, and instruments like the COMRADE may be too broad and indirect to detect changes in the decision-making process. A better alternative is to record conversations between clinicians and patients and observe what is actually happening within that conversation. "

Other information

23) CONSORT: Registration number and name of trial registry

Trial registration: trialregister.nl, Dutch Trial Register (NTR), trial number 10340.

24) CONSORT: Where the full trial protocol can be accessed, if available

http://www.trialregister.nl/trialreg/admin/rctview.asp?TC=3105

25) CONSORT: Sources of funding and other support (such as supply of drugs), role of funders

"This study was supported by the Netherlands Organisation for Health Research and Development (ZonMw); Fonds Psychische Gezondheid; ICT regie; and the Dutch Ministry of Health, Welfare and Sport under the name WEGWEIS (grant number 300020011)."

X26-i) Comment on ethics committee approval

"The medical ethical committee for mental health care (METiGG) assessed the study protocol and judged that the study could be conducted without their approval. "

x26-ii) Outline informed consent procedures

"Informed consent was obtained by research nurses. Patients were provided with an information brochure and they received a phone number and email address of a research assistant who they could contact for further information. A few weeks after the initial information, patients were asked whether they were willing to participated in the trial. "

X26-iii) Safety and security procedures

"Furthermore, an assistant was available by phone for help for three days a week."
In the information brochure contact information was presented. In addition, patients could contact their own case manager.

X27-i) State the relation of the study team towards the system being evaluated

"None declared"
